# Different Types of Mastoid Process Vibrations Affect Dynamic Margin of Stability Differently

**DOI:** 10.3389/fnhum.2022.896221

**Published:** 2022-06-27

**Authors:** Jiani Lu, Haoyu Xie, Jung Hung Chien

**Affiliations:** ^1^Department of Rehabilitation, Shanghai General Hospital, Shanghai Jiao Tong University School of Medicine, Shanghai, China; ^2^Department of Health and Rehabilitation Science, College of Allied Health Professions, University of Nebraska Medical Center, Omaha, NE, United States; ^3^Independent Researcher, Omaha, NE, United States

**Keywords:** stability, vestibular system, gait, vibration, treadmill

## Abstract

The vestibular system is critical for human locomotion. Any deteriorated vestibular system leads to gait instability. In the past decades, these alternations in gait patterns have been majorly measured by the spatial-temporal gait parameters and respective variabilities. However, measuring gait characteristics cannot capture the full aspect of motor controls. Thus, to further understand the effects of deteriorated vestibular system on gait performance, additional measurement needs to be taken into consideration. This study proposed using the margin of stability (MOS) to identify the patterns of dynamic control under different types of mastoid vibrations in walking. This study hypothesized that (1) using the MOS method could facilitate the understanding of another aspect of motor control induced by different types of mastoid vibrations, and (2) applying the mastoid vibrations could induce the asymmetric MOS. Twenty healthy young adults were recruited. Two electromechanical vibrotactile transducers were placed on the bilateral mastoid process to apply different types of vestibular vibrations (bilateral, unilateral, and no vibration). A motion capture system with eight cameras was used to measure the MOSap (margin of stability in the anterior-posterior direction), MOSml (margin of stability in the medial-lateral direction), and respective variabilities. The results were in line with the hypotheses that both bilateral and unilateral mastoid vibrations significantly increased MOSap (*p* = 0.036, *p* < 0.001), MOSml (*p* = 0.012, *p* < 0.001), and respective variabilities *p* = 0.001, *p* < 0.001; *p* = 0.001, *p* < 0.01 when compared to the no vibration condition. Also, significantly larger MOSml (*p* = 0.001), MOSml variability (*p* < 0.023), MOSap (*p* < 0.001), and MOSap variability (*p* = 0.002) were observed under the unilateral vibration condition than that observed under the bilateral vibration condition. The above-mentioned result found that different types of mastoid vibrations affected the MOS differently, suggesting different patterns of control mechanisms under different sensory-conflicted situations. Besides, a significant difference between the dominant and non-dominant legs was observed in MOSml. Moreover, applying the unilateral mastoid vibrations induced a greater symmetric index of MOSml, suggesting that more active control in balance was needed in the medial-lateral than in the anterior-posterior direction.

## Introduction

In the past decades, vibration stimulation has been used to identify the various vestibular disorders, such as patients with unilateral acute vestibular loss (Koo et al., 2011), patients with Meniere's disease (Hong et al., 2007; Marques and Perez-Fernandez, 2012; Lee et al., 2015), patients with the unilateral vestibular or central vestibular deficit (Hamann and Schuster, 1999), and patients with vestibular neuritis (Nuti and Mandalà, 2005). This vibration test was first introduced in 1973 by Lucke to detect abnormality in peripheral vestibular functions (Lücke, 1973). The theory behind this vibration test is that applying the vibration (~100 Hz) directly to the skull or mastoid process induces typical nystagmus, that is, the eyes make uncontrolled movements (Karlberg et al., 2003). Thus, various vestibular disorders can be diagnosed by measuring the frequencies or directions of these eye movements. However, it has been suggested that diagnosing vestibular dysfunction can still be difficult because these vestibular dysfunctions may combine other impairments, such as deficits in vision, proprioception, and musculoskeletal function (Agrawal et al., 2009). Therefore, combining the vibration stimulation with posturography may be a better diagnostic tool for patients with vestibular disorders.

Galvanic vestibular stimulation combined with posturography has been used to manipulate the vestibular afferent signals and further measure the postural control (sway) that an individual counteracts to these artificial vestibular stimuli (Wardman and Fitzpatrick, 2002; Fitzpatrick and Day, 2004; St George and Fitzpatrick, 2011). Additionally, based on different types of galvanic vestibular stimulations, different directional sways could be made. For instance, when the head is facing forward, a binaural bipolar galvanic vestibular stimulation induces body sways in the medial-lateral direction toward the direction of the anode. Besides, a binaural monopolar galvanic vestibular stimulation induces body sways in the anterior-posterior direction (Day et al., 1997). Therefore, by manipulating these different types of galvanic vestibular stimulations with posturography, different types of vestibular disorders can be diagnosed (Tax et al., 2013; Welgampola et al., 2013). However, the use of galvanic vestibular stimulation has its limitations and could bias the outcomes due to the inevitable side effects, such as skin irritations, burns, and discomfort pain underneath and around the stimulus electrodes (Utz et al., 2011). Thus, applying vibration-based vestibular stimulations may be an alternative to eliminate these biases and side effects (Lin et al., 2022). In a previous study (Lin et al., 2022), using vibration-based vestibular stimulations combined with posturography indeed (1) could differentiate postural control patterns according to the aging-related vestibular deterioration, and (2) could also recognize the different types of postural control patterns based on different types of vibration-based vestibular stimulations. Importantly, these different types of sway patterns are calculated by the sway of the center of pressure, which is the most common technology to measure postural control. However, the knowledge of using vibration-based vestibular stimulation for evaluating the dynamic stability through measuring posturography during walking is limited.

The vestibular system is critical for human locomotion (Bent et al., 2004). Specifically, the otolith organs, the saccule, and the utricle can detect the linear acceleration of body movement and stabilize the walking balance (Zangemeister et al., 1991). To our best knowledge, only two studies use the net center of pressure to differentiate the walking patterns, while different types of vibration-based vestibular stimulations are given to healthy young and older adults (Chien et al., 2016, 2017). However, this net center of pressure needs to be calculated by force platforms embedded inside the treadmill belt. Such an instrumented treadmill is generally expensive and cannot be afforded by most research laboratories. Thus, a relatively low-cost measure is needed to fully identify the subtle differences in gait patterns induced by the deteriorated/perturbed vestibular system. Also, a study suggests that only measuring the spatial-temporal gait parameters also may not be sensitive enough to capture the full aspect of the effect of deteriorated sensory systems on gait performance (Herssens et al., 2020). The above-mentioned study further suggests that using the margin of stability (MOS, Herssens et al., 2020) may be an effective measure. The MOS is introduced by Hof et al. (2005). Compared to conventional measures using the center of mass (CoM), this MOS also takes the horizontal velocity of CoM into account. Therefore, this MOS includes three critical components to measure the gait stability: the impulse related to instability, the foot placements related to the base of support (BOS), and the control mechanism related to stability in both the sagittal plane and frontal plane (McAndrew et al., 2011). This MOS has been widely used to measure the gait stability under slip-induced perturbations (Liu et al., 2016; Debelle et al., 2020; Li and Huang, 2022), under the sensory-conflicted perturbations (McAndrew et al., 2011; McAndrew Young et al., 2012; Roeles et al., 2018), in patients with neurological disorders (Tisserand et al., 2018; Lencioni et al., 2021), and under cognitive loading (Raffegeau et al., 2022). Specifically, a larger MOS in the medial-lateral direction (MOSml) is found in post-stroke survivors than controls during treadmill walking (Tisserand et al., 2018). Moreover, a larger MOSml is also observed in the non-paretic side compared to the paretic leg (Tisserand et al., 2018). Additionally, when visual or physical perturbations are given to healthy young adults during treadmill walking, a larger MOSml but a smaller MOS in the anterior-posterior direction (MOSap) were observed (McAndrew et al., 2011). These results seemingly suggest that larger values of MOS require higher demands of control. Also, a larger MOS variability has been observed when encountering the sensory perturbations or suffering neurological disorders, indicating an active control within step-to-step is required (McAndrew et al., 2011). Thus, this study attempted to apply this relatively low-cost MOS method to evaluate the gait performance under different types of vibration-based vestibular stimulations.

In the current study, the main objective was to investigate how different types of mastoid vibrations affected the MOS and its variability in both anterior-posterior and medial-lateral directions. This study hypothesized that (1) using the MOS method may facilitate the understanding of another aspect of motor control induced by different types of mastoid vibrations, and (2) applying the mastoid vibrations could induce the asymmetric MOS, particularly in the medial-lateral direction. Moreover, a larger MOSml would be observed in the dominant leg than in the non-dominant leg when the mastoid vibrations were provided to participants.

## Materials and Methods

### Participants

Twenty healthy young adults participated in this study (10 men and 10 women; 24.55 ± 2.14 years old; walking speed: 1.4 ± 0.2 m/s; body mass: 62.23 ± 13.09 kg; height: 1.67 ± 0.09 m). We excluded participants if they had any neurological disorders, neuropathy due to diabetes, joint injuries, or any accidental falls in the prior year. Importantly, these participants were excluded if they got a score above zero on the dizziness handicap inventory, indicating the potential deteriorations in the vestibular system. The sample size in the current study was used based on our previous published work (Chien et al., 2016). The observed power reached approximately one by recruiting 20 healthy young to calculate the effect of mastoid vestibular vibration on the net center of pressure (Chien et al., 2016). This study was approved by the University of Nebraska Medical Center Institutional Review Board (IRB Protocol # 379-17-EP). All participants were required to agree and sign the informed consent before each data collection began.

### Experimental Setup

**Figure 1:**
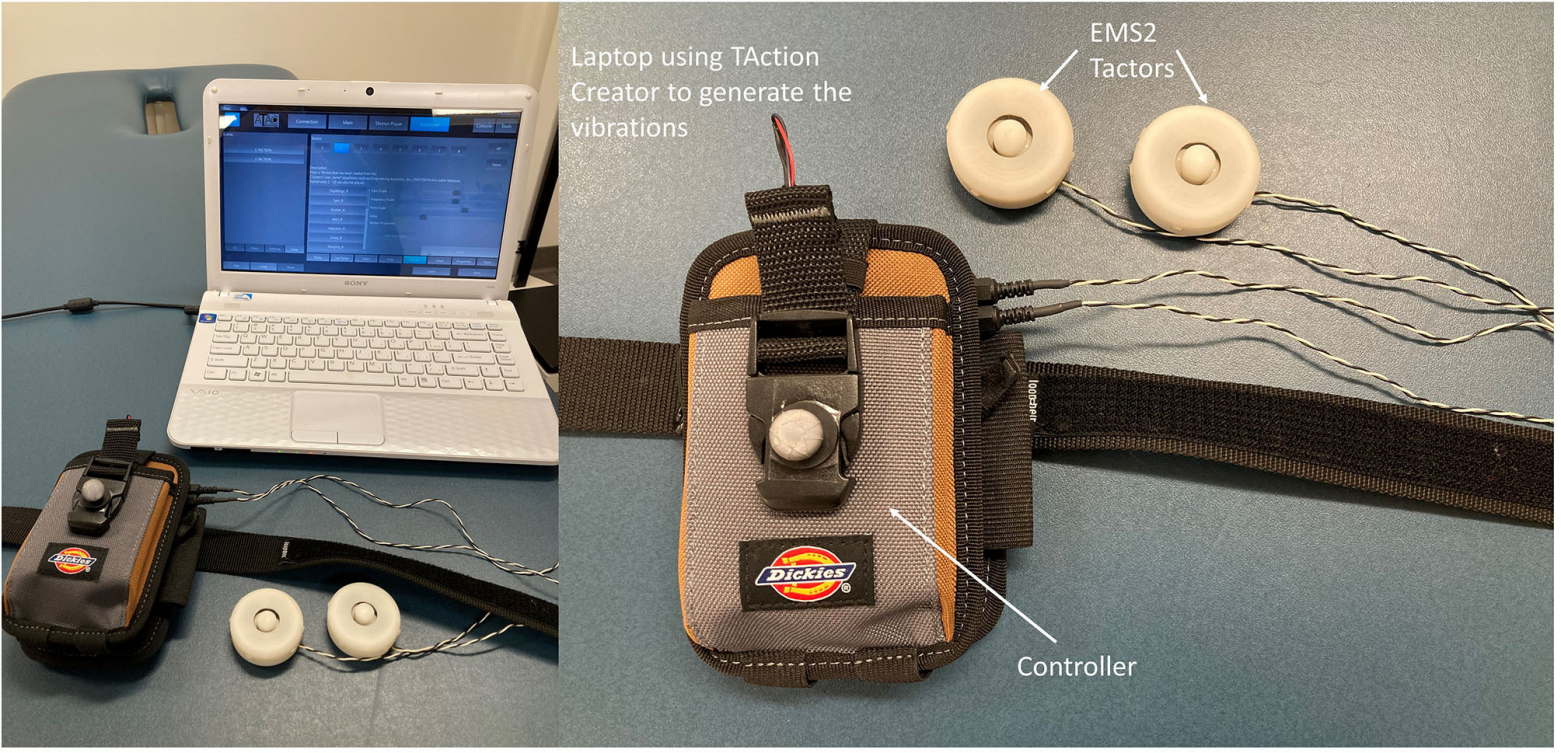
The mastoid vibration device.

A motion capture system with eight high-speed infra-red digital cameras (Qualisys AB, Gothenburg, Sweden) was used to collect three-dimensional gait data at 100 Hz using Qualisys Tracker Manager (QTM) software (Qualisys AB). Twelve retro-reflective markers were placed on the right and left posterior superior iliac spine (PSIS), right and left anterior superior iliac spine (ASIS), each foot (heel and second metatarsal head), each leg (greater trochanter of the femur, lateral epicondyle of the femur, and lateral malleolus). The different types of vestibular vibrations were generated by a mechanical vibrotactile stimulus using two electromechanical vibrotactile transducers (EMS2 tactors; Engineering Acoustics, FL, USA.) that were placed on the mastoid processes bilaterally. These tactors were designed for mounting within a seat or cushion and could produce displacement that allows the vibration to be easily perceived by participants. The EMS2 tactor has a rise time of <25 ms and produces large displacements even when loaded against the mechanical impedance of the body. The maximum peak-to-peak displacement when loaded was 2 mm. The height and weight of tactors were 18.8 mm and 24 g, respectively. The diameter of tactors was 48.5 mm (Figure 1). The frequency of these two mastoid vibrations was set at 100 Hz and was controlled by software (TAction Creator, Engineering Acoustics, FL, USA). The amplitude of supra-threshold vibration was set at 130% of the participant's minimum perceived amplitude (Severini and Delahunt, 2018). To detect the minimum perceived amplitude, an experimenter adjusted the amplitude of vibration through TAction Creator commercial software until participants could perceive while participants stood still. The vibration was started at the 11th second after participants walked on the treadmill. This procedure was to ensure that the unexpected effect of the initial acceleration of the treadmill could not affect the MOS and further could not hinder the “true” effect of different types of vibrations. The active duration of vibration was set at 0.5 s, and the rest duration of vibration was set at 0.5 s to prevent saturation of the vestibular sensation. Three types of mastoid vibrations were given to participants: none, bilateral, and unilateral vestibular vibrations. The unilateral vestibular vibration was only administered through the tactor placed on the left mastoid process to get a consistent outcome between participants. Also, the bilateral vestibular vibration was administrated simultaneously through the tactors placed on bilateral mastoid processes.

### Experimental Protocol

A total of three walking trials were randomly assigned to participants in one visit. We limited the number of trials because this study attempted to understand the effect of the acute vestibular vibrations on the dynamic MOS (Lin et al., 2022). To identify the dominant leg, participants were asked, “What leg did you prefer to kick a soccer ball?” Each walking condition lasted 3 min. A 2-min mandatory rest was given between trials to eliminate the learning effect from the previous trial (Lin et al., 2022). At the end of each trial, the participants were asked if they felt any uncomfortable sensations, such as nausea, vomiting, or dizziness. If they felt any uncomfortable sensation during any walking condition, the experiment was terminated. In addition, none of these participants had experiences with mastoid vibrations. Before the data were recorded, the preferred walking speed needed to be defined for each participant. First, each participant stood on the side of the treadmill without touching the belt and holding the handrail. The treadmill speed (Biodex RTM 600, Shirley NY, USA) then was speeded up to 0.8 m/s. Next, participants stepped on the treadmill belt and began to walk. These young participants were encouraged to walk naturally without holding the handrail. The experimenters then began to ask these young adults, “Is this walking speed similar to the walking speed when you walk around your neighborhood,” this temporary treadmill speed was repeatedly adjusted after 10 s (±0.1 m/s) until participants had an agreement. Then, participants continued walking with a safety lanyard at their preferred walking speed for 5 min for the purpose of familiarization.

### Data Analysis

**Figure 2:**
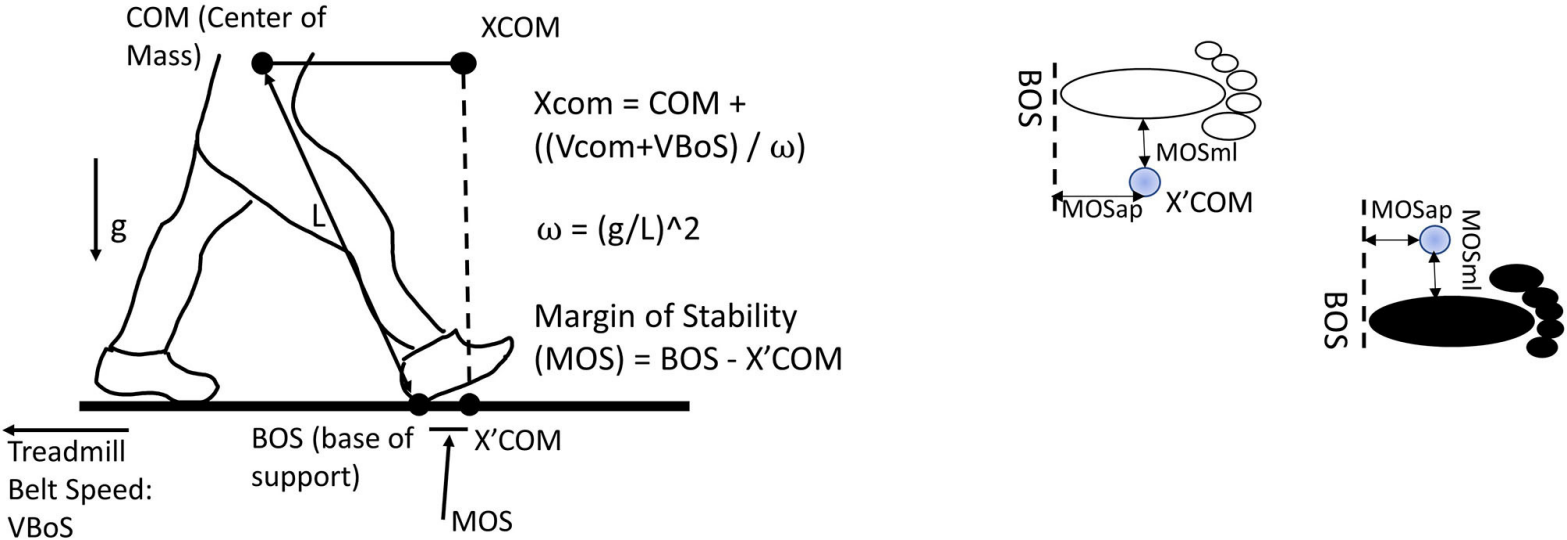
Margin of stability in the anterior-posterior direction (MOSap) was defined as the horizontal distance between the anterior boundary of the base of support (BOS) and the extrapolated center of mass (XCoM). The anterior boundary of the BOS was defined as the position of the heel marker in the anterior-posterior direction. Margin of stability in the medial-lateral direction (MOSml) was defined as the horizontal distance between the lateral boundary of the BOS and the XCoM. The lateral boundary of the BOS was defined as the position of the heel marker in the medial-lateral direction. CoM, center of mass; XCoM, extrapolated center of mass; BOS, base of support; VBOS, velocity of BOS, defined as treadmill belt speed; VCoM, velocity of CoM; MOS, margin of stability; L, the distance from CoM to the heel marker; g, gravity.

Dynamic modified MOS was defined based on Hof's study (Hof et al., 2005) and Süptitz et al.'s (2012) and Fallahtafti et al.'s (2021) studies. First, the CoM was calculated as the average position of markers from the right and left anterior and posterior superior iliac spine (McAndrew et al., 2011). Then, the extrapolated center of mass (XCoM) was calculated as $XCOM=x+\;\frac{\dot{x}+{\dot{x}}_{treadmill}}{\omega_{0}}$, where *x* was the CoM position and ẋ was the CoM velocity, when computing the first derivative of CoM position. ẋ_*treadmill*_ was the treadmill speed. And, $\omega_{o}=\;\sqrt{\frac{g}{l}}$, where *g* = 9.81 m/s^2^ and *l* was the distance from CoM to the heel marker at the initial heel contact (Süptitz et al., 2012; Fallahtafti et al., 2021). The treadmill speed was calculated by an equation: (the distance from heel marker contacted the ground to heel marker off the ground)/(the time from heel contacted the ground to heel off the ground) per step cycle for each leg. Then the base of support (BOS) was defined using heel marker position in the anterior-posterior and medial-lateral directions. The MOSap was calculated as the BOS minus the XCOM in the anterior-posterior direction; therefore, the MOSap should be negative and considered relative-stable in the anterior-posterior direction (McAndrew Young et al., 2012). If the MOSap was positive, it was considered relative-unstable in the anterior-posterior direction (McAndrew Young et al., 2012). The MOSml was calculated as the BOS minus the XCOM in the medial-lateral direction; in contrast, the MOSml should be positive to be considered relative-stable in the medial-lateral direction (McAndrew Young et al., 2012). If the MOSml was negative, it was considered relative-unstable in the medial-lateral direction (McAndrew Young et al., 2012) (Figure 2). It was worth mentioning that “relative-unstable” phase was not necessary to be identified as falls or losing balance in the current study, since these young adults walked with a consistent forward progression (Hof, 2008). Besides, this “relative-unstable” phase in the current study could be defined as a tendency to increase/decrease the value of MOS, which was induced by the vestibular stimulations, in comparison with walking with no vestibular simulation. A total of 200 MOS points were used in this current study, indicating that 100 right initial heel contacts and 100 left initial heel contacts were used. These 200 steps were from step number 101 to step number 200 for each leg. The reason for selecting this interval was to prevent the step-to-step fluctuations caused by speeding up and slowing down at the beginning/end of the trial.

The MOS variability was defined as the standard deviation of all MOS values for a given trial. The initial heel contact was defined when the horizontal heel displacement reached a maximum (Parks et al., 2019). The step length was defined as the traveling distance of the treadmill belt in the anterior-posterior direction from one heel contact to another contralateral heel contact. Also, the step width was defined as the distance between two consecutive heel contacts in the medial-lateral direction. To examine the symmetry of the MOS and its variability induced by different types of vestibular stimulations in both anterior-posterior and medial-lateral directions, the symmetric index was calculated as follows:


$$\begin{array}{l} Symmetric\;Index=\;\frac{X_{DL}-X_{NDL}}{0.5\;\left(X_{DL}+X_{NDL}\right)} \end{array}$$

where x is the dependent variable, DL is the dominant leg, and NDL is the non-dominant leg. A value of zero for the symmetric index indicated a perfect gait symmetry. If the symmetric index of MOS and its variability was positive, the asymmetric patterns were toward the dominant leg and vice versa. Overall, the dependent variables used in this study were MOSap, MOSml, MOSap variability, MOSml variability, step length, step length variability, step width, step width variability, and symmetric index of MOSap, MOSml, MOSap variability, and MOSml variability.

### Statistical Analysis

A Shapiro–Wilk normality test was used to test the normality of each dependent variable, with the alpha value set at 0.05. If the data were normally distributed, a repeated measures ANCOVA with walking speed as a covariate was used to identify the effect of different types of mastoid vibrations on each dependent variable. *Post-hoc* multiple comparisons were corrected by the Bonferroni method. Another two-way repeated measure was used to determine the interaction between the effect of different types of mastoid vibrations and the effect of different legs on MOSap and MOSml. If the data were not normally distributed, a Friedman test was used to investigate the effect of different types of vestibular manipulations on each dependent variable. Wilcoxon signed-rank test was used for pairwise comparisons for each dependent value. The effect size was calculated using the partial eta squared method. The value of 0.138 indicates a large effect size, 0.059 indicates a moderate effect size, and 0.01 indicates a small effect size (Cohen, 1988). This study used G^*^power (URL: http://www.gpower.hhu.de/) to calculate the power. In this study, the η^2^ = 0.09 was selected to calculate the effect size f because this number is between 0.059 for the moderate effect size and 0.138 for the large effect size based on the partial eta squared method. By this calculation, recruiting 19 healthy young adults could reach 80% power for using the repeated measure. This sample size was also based on our previous publication because the results showed a large effect size (Chien et al., 2016), which measures the net center of pressure trajectories under different types of mastoid vibrations.

## Results

### Normality Tests and Effect Size

The alpha values of Shapiro–Wilk test for MOSap, MOSml, MOSap variability, MOSml variability, step length, step length variability, step width, and step width variability were >0.05, indicating that the data were normally distributed. Therefore, a one-way repeated measure was used for the above-mentioned dependent variables. However, the alpha values of Shapiro–Wilk test for values of the symmetric index in MOSap, MOSml, MOSap variability, and MOSml variability were smaller than 0.05, indicating that the data were not normally distributed. Therefore, a Friedman test was used. Partial eta squared values were 0.755 for MOSap, 0.695 for MOSml, 0.750 for MOSap variability, and 0.664 for MOSml variability. These results indicated a large effect size based on the literature (Cohen, 1988).

### The Effect of Mastoid Vibrations on Margins of Stability and Its Variability

**Figure 3:**
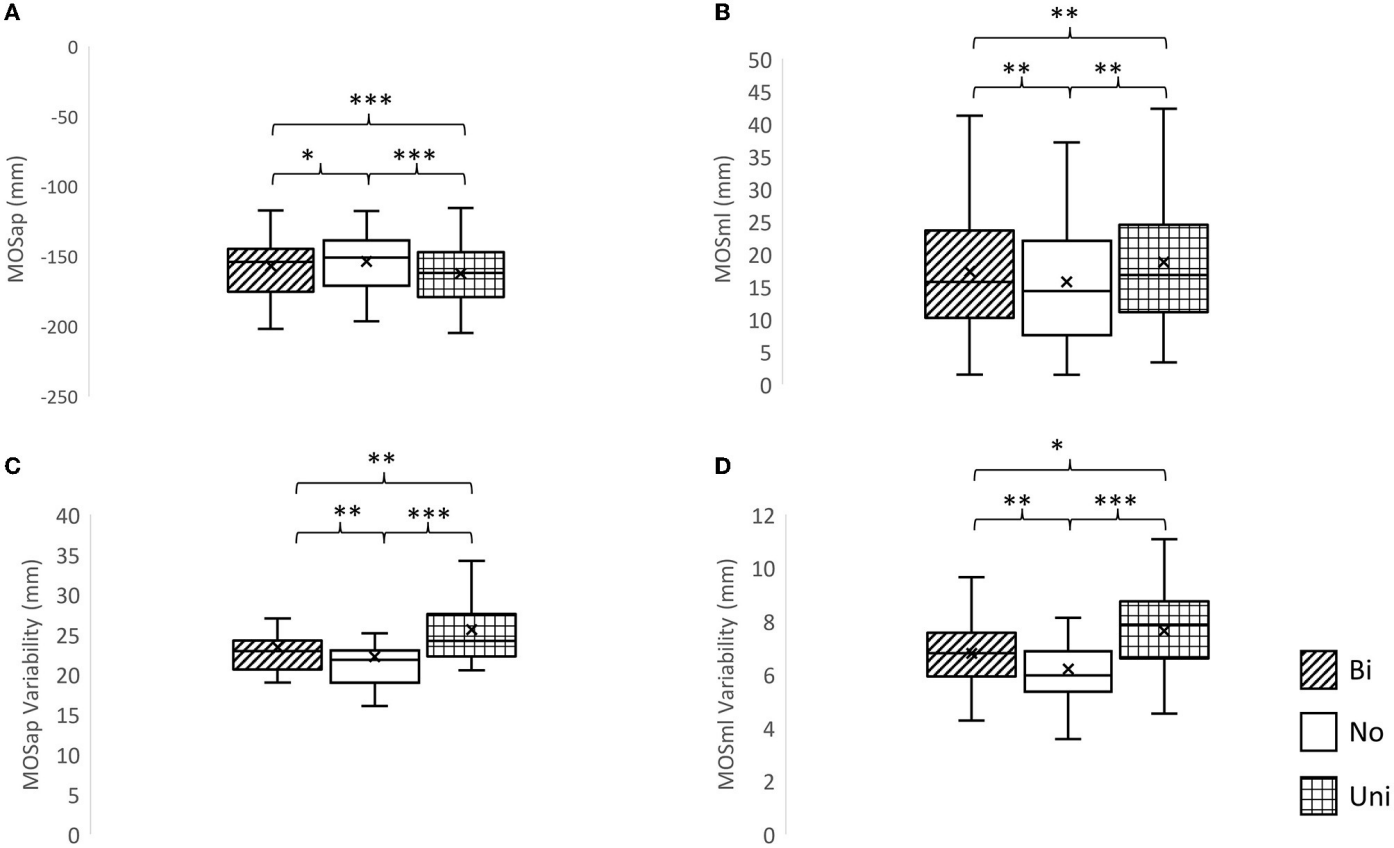
The effects of mastoid vibration on the margin of stability and its variability. **(A)** Mean margin of stability in the anterior-posterior direction (MOSap), **(B)** mean margin of stability in the medial-lateral direction (MOSml), **(C)** variability of MOSap, and **(D)** variability of MOSml. Error bars indicate between-subject standard deviations. *p < 0.05, **p < 0.01, ***p < 0.001, Bi, bilateral mastoid vibration; No, no mastoid vibration; Uni, unilateral mastoid vibration. Symbol x meant the mean value.

A significant effect of mastoid vibration was found in the MOSap (F_2, 22_ = 33.83, *p* < 0.0001), the MOSml (F_2, 22_ = 25.06, *p* < 0.0001), the MOSap variability (F_2, 22_ = 32.98, *p* < 0.0001), and the MOSml variability (F_2, 22_ = 21.71, *p* < 0.0001, Figure 3). The pairwise comparisons indicated that (1) the MOSap was significantly larger (mathematically smaller value in negative value) when either bilateral or unilateral mastoid vibration was given to participants (*p* = 0.036, *p* < 0.001, respectively) in comparison with no vibration was given; (2) the MOSml was significantly larger when either bilateral or unilateral mastoid vibration was given to participants (*p* = 0.0012, *p* = 0.001, respectively) in comparison with no vibration was given; (3) both MOSap and MOSml were significantly larger when unilateral mastoid vibration was given to participants than when bilateral mastoid vibration was given (*p* < 0.001, *p* = 0.001, respectively); (4) For both MOSap variability and MOSml variability, the larger variabilities were observed when either bilateral or unilateral mastoid vibration was given to participants (*p* = 0.001, *p* < 0.001, respectively; *p* = 0.001, *p* < 0.001, respectively) in comparison with no vibration was given. (5) When compared with bilateral mastoid vibration, unilateral mastoid vibration induced higher MOSml variability and MOSap variability (*p* = 0.023, *p* = 0.002).

### The Effect of Dominant Leg on Margins of Stability

A significant interaction between the effect of dominant leg and the effect of vestibular vibration was found in MOSml (F_2, 38_ = 3.805, *p* = 0.031) but not in MOSap (F_2, 38_ = 1.203, *p* = 0.311). The marginal means indicated that MOSml was significantly larger in the dominant leg than in the non-dominant leg (*p* < 0.001). Additionally, pairwise comparisons showed that the values of MOSml were larger in the dominant leg than in the non-dominant leg among the bilateral (*p* **<** 0.001), no (*p* **<** 0.001), and unilateral mastoid vibrations (*p* **<** 0.001). More details are presented in Table 1.

**Table 1:** Summary of right and left MoSap and MoSml (mm) for all participants.

**Leg**	**Condition**					
	**No**	**Bi**	**Uni**	**Effect of VP**	**Effect of DL**	**Interaction**
**MOSap (mm)**						
DL	−159.75 ± 23.03	−154.44 ± 22.85	−164.41 ± 23.91	**F**_**2, 38**_ **=** **39.49;** ***p*** **<** **0.001**	F_1, 19_ = 3.73; *p* = 0.068	NS
NDL	−154.68 ± 23.47	−152.23 ± 23.72	−160.29 ± 26.36			
**MOSml (mm)**						
DL	20.94 ± 11.39	18.51 ± 10.07	22.55 ± 10.16	**F**_**2, 38**_ **=** **23.89;** ***p*** **<** **0.001**	**F**_**1, 19**_ **=** **34.47;** ***p*** **<** **0.001**	**F**_**2, 38**_ **=** **3.81;** ***p*** **=** **0.031**
NDL	13.62 ± 10.76	12.94 ± 10.08	14.89 ± 11.27			

*VP, vestibular perturbation; DL, dominant leg; NDL, non-dominant leg. Bi, bilateral mastoid vibration; No, no mastoid vibration; Uni, unilateral mastoid vibration. NS, not significant*.

*Bold fonts represented the level of significance was reached*.

### The Effect of Different Types of Vibrations on Symmetric Index

**Figure 4:**
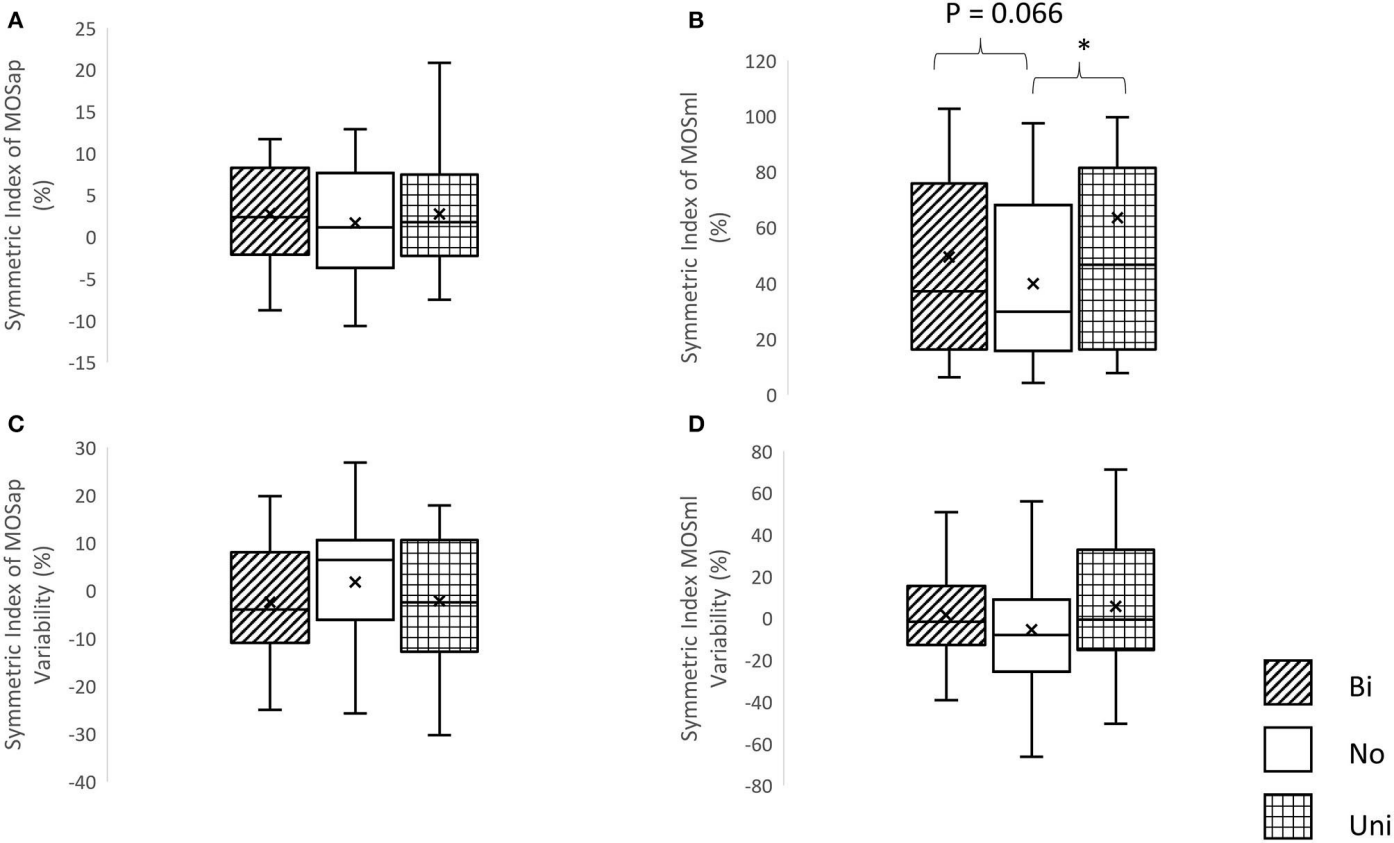
The symmetric index of margin of stability and its variability. **(A)** Symmetric index of margin of stability in the anterior-posterior direction (MOSap), **(B)** symmetric index of margin of stability in the medial-lateral direction (MOSml), **(C)** symmetric index of MOSap variability, and **(D)** symmetric index of MOSml variability, *p < 0.05, Bi, bilateral mastoid vibration; No, no mastoid vibration; Uni, unilateral mastoid vibration. Symbol x meant the mean value.

A significant effect of mastoid vibration was only found in the symmetric index of MOSml (χ^2^ = 7.5, *p* = 0.024). Significantly greater symmetric indices of MOSml were observed in conditions of the unilateral mastoid vibrations (*Z* = −2.352, *p* = 0.019) in comparison with the condition without vibration. More details are shown in Figure 4.

### The Effect of Different Types of Vibration on Spatial Gait Parameters and Respective Variabilities

A significant effect of mastoid vibration was found in the step width (F_2, 38_ = 4.15, *p* = 0.03) and in the step width variability (F_2, 38_ = 27.82, *p* < 0.001). Pairwise comparisons found that applying the unilateral mastoid vibration significantly reduced the step width (*p* = 0.025) and increased the step width variability (*p* < 0.001) compared to the non-vibration condition. Additionally, applying the bilateral mastoid vibration significantly increased the step width variability (*p* < 0.001) compared to the non-vibration condition. More detail are presented in Table 2.

**Table 2:** The mean values of spatial-temporal gait parameters and respective gait variabilities.

**Step length (MM)**		**VP**	**Bi vs. No**	**Bi vs. Uni**	**No vs. Uni**
Bi	567.41 (60.21)	F = 1.017; *p* = 0.378	NA	NA	NA
No	577.43 (55.92)				
Uni	567.24 (58.36)				
**Step width (MM)**			**BI vs. NO**	**BI vs. UNI**	**NO vs. UNI**
Bi	119.79 (26.61)	**F** **=** **4.145;** ***p*** **=** **0.030**	NS	NS	***p*** **=** **0.025**
No	122.98 (26.73)				
Uni	116.83 (26.74)				
**Step length variability (MM)**			**BI vs. NO**	**BI vs. UNI**	**NO vs. UNI**
Bi	21.73 (4.44)	F = 2.479; *p* = 0.107	NA	NA	NA
No	21.26 (5.91)				
Uni	20.96 (4.59)				
**Step width variability (MM)**			**BI vs. NO**	**BI vs. UNI**	**NO vs. UNI**
Bi	20.06 (3.50)	**F** **=** **27.816;** ***p*** **<** **0.001**	***p*** **=** **0.001**	NS	***p*** **<** **0.001**
No	17.09 (2.78)				
Uni	19.99 (3.22)				

*Bi, bilateral mastoid vibration; No, no mastoid vibration; Uni, unilateral mastoid vibration; NA, not available; NS, not significant; VP, vestibular perturbation*.

*Bold fonts represented the level of significance was reached*.

### The Interaction Between Effect of Walking Speed on MOS and Respective Variabilities, and Spatial Gait Parameter and Respective Variabilities

The interaction between the walking speed as a covariate and the MOSap (F_16, 22_ = 1.065, *p* = 0.437), the MOSml (F_16, 22_ = 0.841, *p* = 0.634), the MOSap variability (F_16, 22_ = 0.955, *p* = 0.529), the MOSml variability (F_16, 22_ = 1.330, *p* = 0.263), the step length (F_16, 22_ = 0.766, *p* = 0.704), the step length variability (F_16, 22_ = 1.279, *p* = 0.291), the step width (F_16, 22_ = 2.049, *p* = 0.059), and the step width variability (F_16, 22_ = 0.882, *p* = 0.579). More details are presented in Table 3.

**Table 3:** The interaction between the effect of mastoid vibration and the effect of walking speed as a covariate (MV × WS) in MOSap, MOSml, MOSap variability, MOSml variability, step length, step variability, step width, or step width variability.

	**MV × WS**		**WS**	
Mosap	F = 1.065	*p* = 0.437	F = 2.061	*p* = 0.132
MOSml	F = 0.841	*p* = 0.634	F = 0.330	*p* = 0.937
MOSap Variability	F = 0.955	*p* = 0.529	F = 0.276	*p* = 0.961
MOSml Variability	F = 1.330	*p* = 0.263	F = 0.322	*p* = 0.941
Step Length	F = 0.766	*p* = 0.704	F = 0.820	*P* = 0.602
Step Length Variability	F = 1.279	*p* = 0.291	F = 1.148	*P* = 0.405
Step Width	F = 2.049	*p* = 0.059	F = 1.767	*P* = 0.188
Step Width Variability	F = 0.882	*p* = 0.579	F = 2.154	*P* = 0.119

*MV, mastoid vibrations; WS, walking speed*.

## Discussion

This study attempted to understand how MOS and respective variability changed when vestibular vibrations were given to healthy young adults. The results had agreements with our hypotheses that (1) using the MOS method provided another aspect of motor controls induced by different types of mastoid vibrations, and (2) a larger MOS in the medial-lateral direction was observed in the dominant leg than in the non-dominant leg.

### Using MOS Measure Could Understand Another Aspect of Motor Controls Induced by Different Types of Mastoid Vibrations

Gait parameters have been used to assess the effect of vestibular stimulation on gait characteristics and respective variabilities in healthy young adults (Wuehr et al., 2016b) and in patients with bilateral vestibulopathy (Wuehr et al., 2016a). These studies found that applying the noisy galvanic vestibular stimulation significantly reduced the stride length variability but found no changes in the stride length in patients with bilateral vestibulopathy. However, for healthy young adults, such a vestibular stimulation had no impact on the gait asymmetry, the stride length, and the stride length variability (Wuehr et al., 2016b). Therefore, using the gait characteristics and respective variabilities as measures may only provide one aspect of motor control under the vestibular perturbations. On the contrary, in the current study, using the MOS measure might further help in the understanding of another aspect of motor control induced by different types of mastoid vibrations. Unsurprisingly, larger MOS in both anterior-posterior and medial-lateral directions were observed when either bilateral or unilateral mastoid vibrations were given compared to no vibration was given. Moreover, applying the unilateral mastoid vibration during walking increased the MOS and the MOS variability in both anterior-posterior and medial-lateral directions than applying the bilateral mastoid vibration. These results demonstrated the different types of motor controls under different types of mastoid vibrations. Also, these results agreed with previous studies that providing the visual or platform-induced perturbations increased the MOS in the medial-lateral direction (McAndrew et al., 2011). This observation could be explained by the sensory reweighting hypothesis (Peterka, 2002). This hypothesis indicated that each sensory channel contributed different weights of environmental information to the central nervous system (CNS), and then CNS summed all weighted information to produce an appropriate movement. If a sensory signal was weak or perturbed, the CNS weighted heavily on other reliable sensory systems to generate appropriate postural behaviors. However, during this process of sensory reweighting from unreliable to reliable sensory system, the increase/decrease of MOS could be observed to maintain dynamic gait stability (McAndrew et al., 2011). Based on the formula of MOS (Süptitz et al., 2012; Fallahtafti et al., 2021), two factors could lead to a larger MOS: (1) the BOS was toward COM or (2) XCoM was far away from COM. In the current study, the spatial gait parameter supported that a larger MOSap was attributed to the BOS being toward the COM (shorter step length) when both types of mastoid vibrations were applied compared to the condition of no vibration. This observation also was supported by the increase in cadence in patients with vestibulopathy (Krebs et al., 2002; Dale, 2012). Based on the equation: step length = walking velocity/cadence (Dale, 2012), higher cadence led to shorter step length if the walking speed was constant (treadmill speed was constant among conditions). Then, the shorter step length led to the BOS being toward the COM, resulting in a larger MOSap in the present study. Similarly, a larger MOSml due to the mastoid vibrations might also be attributed to the BOS being toward the COM (smaller step width) in the current study.

Interestingly, while applying the vibrations to the mastoid process during walking, the increased rates of MOS were different in the anterior-posterior direction (MOSap, Bilateral vs. No: 2.41%, Unilateral vs. No: 5.70%) than in the medial-lateral direction (MOSml, Bilateral vs. No: 10.91%, Unilateral vs. No: 18.87%). This finding could be explained by the active control hypothesis (Bauby and Kuo, 2000). According to the above-mentioned hypothesis (Bauby and Kuo, 2000; O'Connor and Kuo, 2009; Hu and Chien, 2021), when the somatosensory and/or visual systems were perturbed, the lateral control had a higher priority than fore-aft control (O'Connor and Kuo, 2009; Hu and Chien, 2021), indicating that stabilizing the balance in the medial-lateral direction was much difficult than in the anterior-posterior direction due to the biomechanical structure of the human body (Bauby and Kuo, 2000). Similarly, when applying mastoid vibration, the level of active control was higher in the medial-lateral direction than in the anterior-posterior direction. Another interesting observation was that applying the unilateral mastoid vibration led to higher MOSap and MOSml than applying the bilateral mastoid vibration. The current finding challenged the hypothesis of the Bayesian framework, indicating that if more sensory signals were available, a more precise estimation could be made. Subsequently, more appropriate postural can be corrected (Chien et al., 2017). One possible rationale might be that this unilateral mastoid vibration induced vestibular illusions like galvanic vestibular stimulation (Wardman et al., 2003). In Wardman et al.'s (2003) study, when applying a long galvanic vestibular stimulation during standing (400 ms duration similar to 500 ms duration in this study), the illusions like “tilt” or “spin” toward the side of the vestibular stimulation could be perceived, and the body moved to opposite direction where vestibular stimulation was provided. Moreover, similar findings were observed when humans deviated from the straight pathway but still thought they walked straightly under unilateral galvanic vestibular stimulation (Fitzpatrick et al., 1999). Therefore, we speculated that this vestibular illusion caused by unilateral mastoid vibrations required extra effort for CNS to process and eventually increase the MOSml, particularly on the side of the dominant leg (more discussion can be found in Using Dominant Leg to Control the Margin of Stability in the Medial-Lateral Direction). Also, a non-yet-certified peer-reviewed study (Kazanski et al., 2021) proposed a probability of instability to predict the instability risk related to MOSml. This previous study had an agreement with the current study that a larger mean MOSml could be a sign of increased risk of lateral instability.

It has been shown that only measuring the means of MOSap and MOSml was not enough to understand the entire aspect of control mechanisms (McAndrew et al., 2011). Means of MOSap and MOSml could not demonstrate how each step related to the next step because mean MOSap and MOSml only quantified overall stability over each walking condition. Quantifying step-to-step MOSap and MOSml variabilities could investigate how individuals control their step-to-step stability under different types of mastoid vibrations like other studies (Hausdorff et al., 1995; Gates et al., 2007). In the current study, the MOSap and MOSml variabilities were significantly larger when the vestibular system was perturbed, and similar changes have also been found during other sensory perturbations in other studies (McAndrew et al., 2011; McAndrew Young et al., 2012; Roeles et al., 2018). In other words, the step-to-step stability required high demands in controls to actively resolve the sensory-conflicted conditions in both anterior-posterior and medial-lateral directions (Hu and Chien, 2021) when either bilateral or unilateral vestibular vibration was provided. Additionally, in the current study, step-to-step stability required even higher demands in controls to counter the instability (Kazanski et al., 2021) induced by the above-mentioned vestibular illusion in the medial-lateral direction when unilateral vestibular vibration was provided than when bilateral vestibular vibration was provided.

### Using Dominant Leg to Control the Margin of Stability in the Medial-Lateral Direction

In regular walking, investigating MOSap on one leg or mean MOSap from both legs was sufficient for determining representative characteristics (Süptitz et al., 2012). The direct evidence was that when instructing a healthy young adult to walk at five different speeds on a treadmill (from 1.0, 1.2 … to 2.0 m/s), the symmetric index of MOSap did not change with changing speeds, suggesting that investigating the MOSap in one leg was sufficient (Süptitz et al., 2012). However, this above-mentioned study (Süptitz et al., 2012) had one fundamental flaw, that is, the MOSml was neglected. The current study confirmed that the symmetric index of MOSap did not change regardless of whether the mastoid vibrations were applied or not. Also, the symmetric index of MOSap was 2.77% for the bilateral mastoid vibration condition, 1.65% for the no mastoid vibration condition, and 2.72% for the unilateral mastoid vibration, indicating a symmetric gait pattern because the symmetric index was below 8% (Süptitz et al., 2012). However, the current study found that applying either bilateral or unilateral mastoid vibration significantly increased the MOSml than no vibration condition. Also, larger MOSml was found in the dominant leg than in the non-dominant leg in all the conditions. This result was similar to Tisserand's study in that a larger MOSml was observed in the non-paretic side compared to the paretic leg (Tisserand et al., 2018). In other words, the dominant leg could potentially play a leading role in making a pattern/trajectory for controlling in advance. Then, the non-dominant leg followed the control mechanism that the dominant leg had been made. Due to the perturbed vestibular system in the current study, the somatosensory/proprioceptive sensor systems might increase gain in the dominant leg (Chien et al., 2016), resulting in the increase of MOSml. We speculated the purpose of this dominant-leg control mechanism was to simplify/optimize the control and then to transfer the learned trajectory to the non-dominant leg to prevent slipping under perturbed sensory situations.

### Preferred Walking Speed Among Young Adults Did Not Affect the MOSap and MOSml

Did walking speeds affect the MOSap and MOSml? This question was controversial. In McCrum et al. (2019)'s study, the MOSap demonstrated a negative correlation between 1.0 and 1.6 m/s, indicating that faster walking speed caused smaller MOSap. However, in Süptitz et al.'s (2012) study, the walking speed seemingly had a very weak correlation (between 1.0 and 1.6 m/s) with MOSap. A more negative MOSap was observed as the walking speed increased. In other words, a more negative MOSap indicated that the margin was exceeded, and the extrapolated COMap was outside of the BOS (Fallahtafti et al., 2021). In the current study, we defined that a more negative MOSap indicated a larger MOSap. Additionally, the MOSml decreased with increasing walking speed, implying a negative correlation in the above-mentioned speed range (Fallahtafti et al., 2021). In the current study, when adding the walking speed as a covariate, the results showed no interactions between walking speed and mastoid vibration in the MOSap, MOSml, MOSap variability, MOSml variability, step length, step length variability, the step width, or the step width variability. Moreover, no major effect of walking speed was found on these above-mentioned dependent variables. The above-mentioned results suggested that the changes in MOSap, MOSml, MOSap variability, or MOSml variability were purely induced by the mastoid vibrations and did not interfere with the walking speed. This study supported the findings of Süptitz et al.'s (2012) study and speculated that the small range of walking speed (1.14–1.79 m/s) in this study might not interfere with the margin of stability.

### A Major Concern About Using the Simplified Marker-Setting for Margin of Stability

A concern raised in this current study was the accuracy of the simplified marker-setting for calculating MOS. In Havens et al.'s (2018) study, the effect of different types of marker-setting on MOS was investigated during three major locomotor behaviors: straight walking through 8 m walkway, walking 4 m and making a 90° to the left, and walking 4 m and make a 90° to the right. Also, the golden standard marker-setting was the full-body marker-setting, where the weighted average of each of the 15 segments' center of mass was used to calculate the whole-body center of mass. The simplified makers-setting was the pelvis average model, which was also used in the current study (see Methods). In this previous study, the results found that the errors produced by the above-mentioned simplified marker-setting relative to the gold standard maker-setting were ~-5 and 5% during straight walking, which might exceed the of the statistical differences (Havens et al., 2018), indicating a potential issue for interfering with the “true” outcomes in the current study. Specifically, this previous study indicated that using the pelvis average model might overestimate the COM distance and velocity, contributing to MOS error even walking straight. Therefore, this concern might be the primary limitation for losing the accuracy of MOS measures in the current study. However, walking on the treadmill differed from walking overground (Hollman et al., 2016). In general, walking on the treadmill has relatively limited degrees of freedom in body movement compared to walking overground. Would similar accuracy issues of MOS measure based on simplified marker-setting occur during the treadmill walking? To our best knowledge, this information was absent. This current study strongly suggested that future studies should resolve this accuracy issue by investigating the effect of different types of marker-setting on the treadmill and overground walking.

## Limitations and Future Applications

There were a couple of limitations in the current study. First, the sample size of this study was set as a moderate effect size, indicating more participants may need to be recruited in the future. We decided to use the current sample size based on our previous publications, which was to investigate the effect of mastoid vibrations on the net center of pressure (Chien et al., 2016), and also was based on the predicted sample size calculation through G^*^Power. Nevertheless, these current partial eta squared values indicated a large effect size in this study. Second, different frequencies and amplitudes of mastoid vibrations might affect MOS differently. In the current study, the combination of frequency and amplitude was based on previous literature. Investigating the effects of different combinations of frequency and amplitude on MOS is needed to build a full aspect of understanding in the application of mastoid vibrations.

## Conclusion

To our best knowledge, this study was the first to use MOS as a measure to determine the motor controls induced by different types of mastoid vibrations. Although the simplified marker-setting might be a potential factor for interfering with the “true” outcomes, the observations in the current study suggested that different types of mastoid vibrations affected MOS differently. It is worth mentioning that applying both types of mastoid vibrations increased the symmetric index in the medial-lateral direction, indicating the dominant leg might play a major role in leading the walk under the sensory-conflicted conditions.

## Data Availability Statement

The original contributions presented in the study are included in the article/Supplementary Material, further inquiries can be directed to the corresponding author/s.

## Ethics Statement

The studies involving human participants were reviewed and approved by University of Nebraska Medical Center Institutional Review Board. The patients/participants provided their written informed consent to participate in this study.

## Author Contributions

JC and JL reviewed previous literature and generated an idea to write this manuscript. JC designed and carried out the experiments. All authors contributed to the article, wrote the main text, and approved the submitted version.

## Conflict of Interest

The authors declare that the research was conducted in the absence of any commercial or financial relationships that could be construed as a potential conflict of interest.

## Publisher's Note

All claims expressed in this article are solely those of the authors and do not necessarily represent those of their affiliated organizations, or those of the publisher, the editors and the reviewers. Any product that may be evaluated in this article, or claim that may be made by its manufacturer, is not guaranteed or endorsed by the publisher.
